# Mild hypothermia attenuates ischaemia/reperfusion injury: insights from serial non-invasive pressure–volume loops

**DOI:** 10.1093/cvr/cvad028

**Published:** 2023-02-03

**Authors:** Jonathan Berg, Robert Jablonowski, David Nordlund, Daniel Ryd, Einar Heiberg, Marcus Carlsson, Håkan Arheden

**Affiliations:** Clinical Physiology, Department of Clinical Sciences LundFaculty of Medicine, Lund University, Box 117 221 00 Lund, Sweden; Skåne University Hospital, Carl-Bertil Laurells gata 9, 214 28 Malmö, Sweden; Syntach AB, Lund, Sweden; Skåne University Hospital, Carl-Bertil Laurells gata 9, 214 28 Malmö, Sweden; Skåne University Hospital, Carl-Bertil Laurells gata 9, 214 28 Malmö, Sweden; Skåne University Hospital, Carl-Bertil Laurells gata 9, 214 28 Malmö, Sweden; Skåne University Hospital, Carl-Bertil Laurells gata 9, 214 28 Malmö, Sweden; Skåne University Hospital, Carl-Bertil Laurells gata 9, 214 28 Malmö, Sweden; Skåne University Hospital, Carl-Bertil Laurells gata 9, 214 28 Malmö, Sweden

**Keywords:** Hypothermia, induced, Acute myocardial infarction, Ischaemia, Reperfusion injury, Pressure–volume loops

## Abstract

**Aims:**

Mild hypothermia, 32–35°C, reduces infarct size in experimental studies, potentially mediating reperfusion injuries, but human trials have been ambiguous. To elucidate the cardioprotective mechanisms of mild hypothermia, we analysed cardiac performance in a porcine model of ischaemia/reperfusion, with serial cardiovascular magnetic resonance (CMR) imaging throughout 1 week using non-invasive pressure–volume (PV) loops.

**Methods and results:**

Normothermia and Hypothermia group sessions (*n* = 7 + 7 pigs, non-random allocation) were imaged with Cardiovascular magnetic resonance (CMR) at baseline and subjected to 40 min of normothermic ischaemia by catheter intervention. Thereafter, the Hypothermia group was rapidly cooled (mean 34.5°C) for 5 min before reperfusion. Additional CMR sessions at 2 h, 24 h, and 7 days acquired ventricular volumes and ischaemic injuries (unblinded analysis). Stroke volume (SV: −24%; *P* = 0.029; Friedmans test) and ejection fraction (EF: −20%; *P* = 0.068) were notably reduced at 24 h in the Normothermia group compared with baseline. In contrast, the decreases were ameliorated in the Hypothermia group (SV: −6%; *P* = 0.77; EF: −6%; *P* = 0.13). Mean arterial pressure remained stable in Normothermic animals (−3%, *P* = 0.77) but dropped 2 h post-reperfusion in hypothermic animals (−18%, *P* = 0.007). Both groups experienced a decrease and partial recovery pattern for PV loop-derived variables over 1 week, but the adverse effects tended to attenuate in the Hypothermia group. Infarct sizes were 10 ± 8% in Hypothermic and 15 ± 8% in Normothermic animals (*P* = 0.32). Analysis of covariance at 24 h indicated that hypothermia has cardioprotective properties incremental to reducing infarct size, such as higher external power (*P* = 0.061) and lower arterial elastance (*P* = 0.015).

**Conclusion:**

Using non-invasive PV loops by CMR, we observed that mild hypothermia at reperfusion alleviates the heart’s work after ischaemia/reperfusion injuries during the first week and preserves short-term cardiac performance. This hypothesis-generating study suggests hypothermia to have cardioprotective properties, incremental to reducing infarct size. The primary cardioprotective mechanism was likely an afterload reduction acutely unloading the left ventricle.

## Graphical Abstract

**Graphical Abstract:**
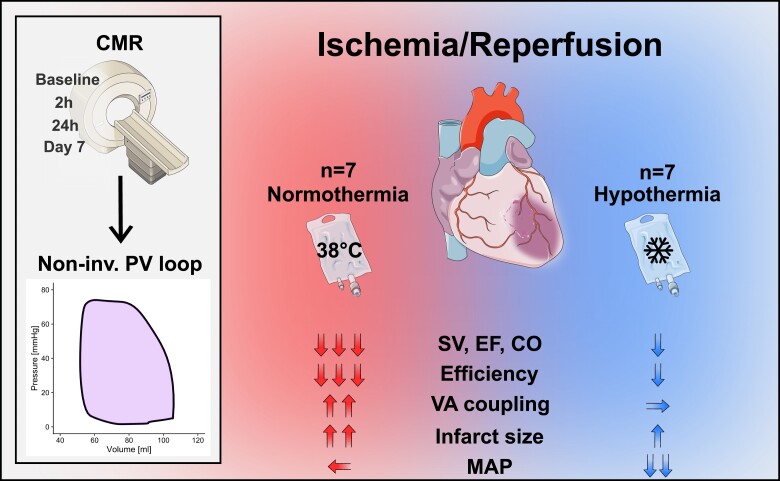

## 1. Introduction

Myocardial infarction (MI) is a significant cause of heart failure globally.^1^ While most MIs are nonfatal, many patients suffer from additional infarctions, angina, and progress towards heart failure. Standard treatment for MI is the timely opening of the culprit artery by catheter intervention to reperfuse the ischaemic myocardium. Reperfusion, however, leads to an additional myocardial injury known as reperfusion injury.

Mild hypothermia, 32–35°C, at reperfusion has been shown to reduce infarct size in several preclinical studies^2,3^ and has been proposed as a treatment to alleviate reperfusion injury. Though preclinical results are promising, human randomized trials in patients with ST-elevation myocardial infarction (STEMI) have been ambiguous,^4–7^ and further efforts to elucidate the potential benefits of hypothermia in MI are ongoing.^8^ Typical endpoints for such trials are infarct size measured by late gadolinium enhancement (LGE) images from cardiac magnetic resonance (CMR).^9,10^ While it is agreed that infarct size is essential for prognosis, this endpoint may lead to overlooking global effects on cardiac function, such as those resulting from cardiac inflammation,^11,12^ catecholamine stress,^13^ or stunning.^14^ Moreover, our understanding of the cardioprotective effects of mild hypothermia after ischaemia and reperfusion remains incomplete due to a lack of studies serially investigating the heart’s function throughout the subacute to long-term phases.

Pressure–volume (PV) loops can be acquired by inserting a conductance catheter in the heart to characterize cardiac performance concerning cardiac energy and mechanics. They have predominantly been studied experimentally^15–17^ as their invasive nature limits their clinical applications. By measuring the pressures and volumes during cardiac pumping, important information regarding the heart’s contractility, energetic efficiency, and interaction with the arterial load is available. As a result, minimally non-invasive methods to derive PV loops have been proposed to bridge the gap in clinical applicability,^18,19^ including one from our group.^20,21^

We aimed to use a porcine model of ischaemia/reperfusion followed by serial imaging for 1 week to elucidate the cardioprotective effects of mild hypothermia concerning cardiac energy and mechanics. Our specific questions were (i) how does mild hypothermia impact cardiac energy mechanics compared with normothermia; (ii) does mild hypothermia provide cardiac protection incremental to a reduction in infarct size; and (iii) how does this newly developed method to acquire PV loops non-invasively by CMR^20,21^ help characterize some of the cardioprotective mechanisms involved?

## 2. Methods

### 2.1 Experimental protocol

This study was performed in accordance with the guidelines from Directive 2010/63/EU of the European Parliament on the protection of animals used for scientific purposes. The study was approved by the Swedish Agricultural Board and the Regional Ethics Committee for animal experiments (registration number: 5.8.18-11702/2019). Normothermia and Hypothermia groups of seven Landrace pigs (40 ± 3 kg, ∼3 months old) in each group were included by non-random allocation. All animals received the same pre-, peri-, and post-operative medications. Pre-medication subsumed a mixture of 30 mg/kg Ketamine (Ketaminol, Intervet, Danderyd, Sweden), 5 mg/kg Midazolam (Dormicum, Roche AB, Stockholm, Sweden), and 0.02 mg/kg Atropine (Atropin, Mylan, Stockholm, Sweden) by an intramuscular injection. Anaesthesia was maintained by a mixture of 2–5% Isoflurane (Isoflurane, Baxter Medical AB, Kista, Sweden), delivered by an inhalation anaesthetic conserving device (AnaConDa, Sedana Medical, Sweden), and oxygen 2–5 L/min. Femoral arterial and venous access was obtained through the Seldinger technique. After transportation in an animal carriage in the lateral decubitus position, baseline CMR imaging for assessment of ventricular function was carried out using a 1.5 T scanner (MAGNETOM Aera, Siemens Healthcare GmbH, Erlangen, Germany). The animals were mechanically ventilated in a supine position during surgical preparation and imaging. After baseline imaging, the animals were transported to a fluoroscopy suite and connected to an intravascular temperature management system (Thermogard XP, ZOLL Circulation, CA, USA) to maintain the baseline body temperature at 38°C.

**Figure 1:**
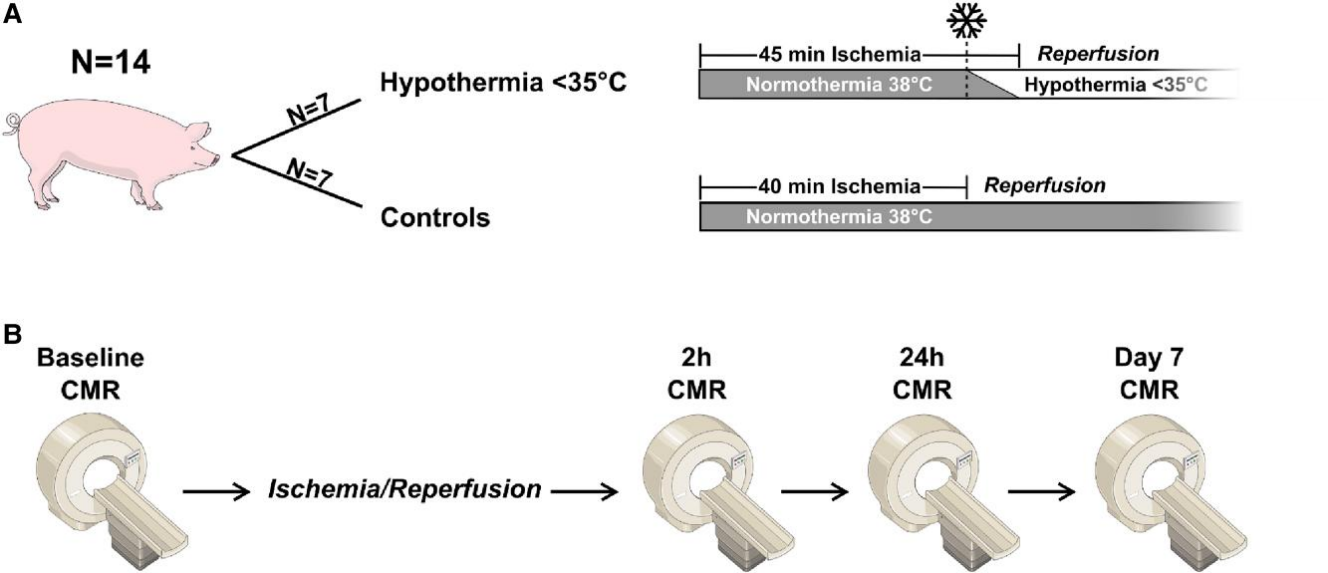
Illustration of the study design, including the ischaemia/reperfusion protocol (*A*) and timing of the CMR sessions (*B*).

Both the Hypothermia and Normothermia groups underwent a normothermic ischaemic period at baseline temperatures for 40 min (*Figure 1A*). Ischaemia was initiated by balloon occlusion of the mid-portion of the left anterior descending artery (see Supplementary material online, *Figure S1*). At 40 min in the Hypothermia group, however, rapid cooling began by infusion of 1 L near zero-degree Ringer’s Acetate and reperfusion ensued 5 min later. A fluid challenge of 1 L ∼37°C Ringer’s Acetate was also administered 5 min before reperfusion in the Normothermia group at 35 min. A central temperature reduction to <35°C before reperfusion was considered successful. The intravascular temperature management system actively maintained the low temperature for 30 min after reperfusion to prevent the rapid temperature rebound phase. A target temperature of at most 35° was chosen since it has been suggested as an optimal balance between clinical feasibility and efficacy of cardioprotection.^4^ Time under ischaemia of 40 and 45 min, respectively, was chosen first to let ∼50% of the myocardium at risk be infarcted^22^ and second to allow both groups to undergo the same length of normothermic ischaemia. This protocol follows the same ischaemia/reperfusion procedure as the hypothermia experiments by Götberg *et al.*^2,23^ and these studies, in combination with pilot experiments, show that cold fluid infusion is required for adequate cooling efficacy.

The animals were moved back into the CMR scanner after the ischaemia/reperfusion period. A second imaging session ensued, 2 h after reperfusion, that included sequences to assess oedema and infarct in addition to ventricular function. After the second imaging session, the animals were transported back to the animal facility and awoken. Two more imaging sessions (at 24 h and 7 days) were carried out with identical anaesthetic protocols (*Figure 1B*). Before termination, an intravenous injection of 0.2 mmol/kg gadolinium contrast agent (Dotarem, Guerbet, Roissy, France) was administered and allowed to circulate for 15 min. The termination was achieved by an intravenous injection of Pentobarbital (250 mg/kg, Euthasol vet, Virbac, Kolding, Denmark). The heart was explanted, and the right ventricle and both atria were trimmed off. The left ventricle (LV) was then imaged *ex vivo* using a T1-weighted sequence. After that, the heart was sliced and stained with triphenyl tetrazolium chloride (TTC) and photographed.

### 2.2 CMR imaging

For baseline imaging, short-axis and long-axis views of the LV were acquired with a balanced steady-state free precession (bSSFP) cine sequence. For post-infarct imaging, a contrast-enhanced balanced SSFP (CE-SSFP) cine sequence, covering both short- and long-axis views, was acquired to quantify ventricular function and estimate myocardium at risk from oedema.^24^ Cine images were acquired with retrospective electrocardiogram gating at end-expiratory breath holds. Typical spatial resolutions were 1.5 × 1.5 × 8 mm with no slice gap and 25 reconstructed time frames. Standard parameters were repetition time (TR) 2.7 ms; echo time (TE) 1.2 ms; flip angle 60°; and field of view (FOV) 270 × 320 mm. LGE images were collected to visualize infarct size *in vivo*. The LGE sequence used the following parameters: TR 2.8 ms; TE 1.2 ms; flip angle 50°; FOV 159 × 154 mm; slice thickness 8 mm. After termination, the LV was imaged *ex vivo* with a high-resolution T1-weighted sequence (0.5 mm isotropic voxel size; TR 20 ms; TE 3.6 ms; flip angle 70°; slice thickness 2.5 mm) for detailed infarct quantification.

### 2.3 Data analysis

**Figure 2:**
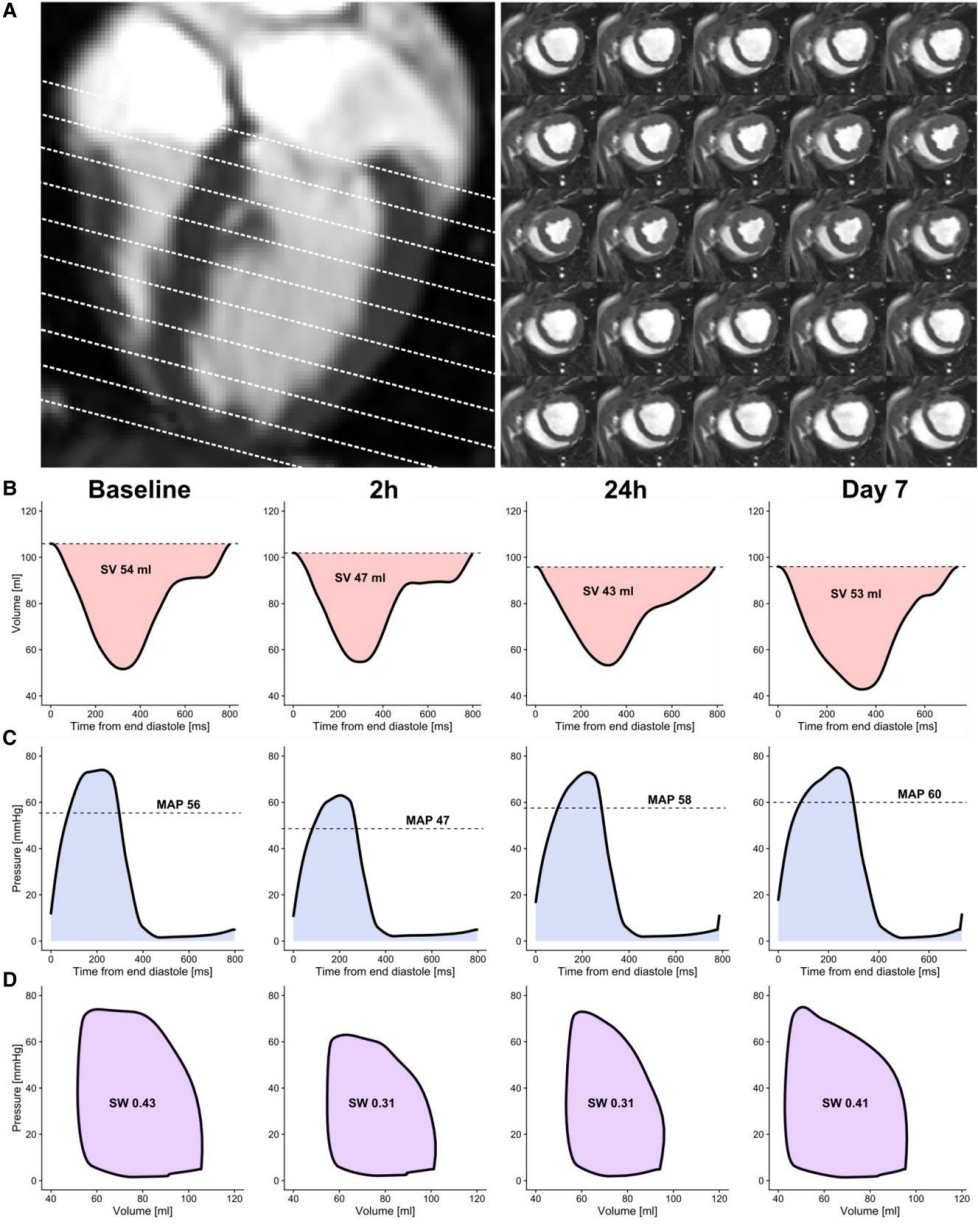
A typical example from the Normothermia group. The basis for the non-invasive PV loops is ventricular volumes plotted against ventricular pressure. Short-axis cine images covering the LV were delineated over the cardiac cycle (*A*). The resulting left ventricular volume curves at each time point (*B*) and the estimated intraventricular pressure curves (*C*) are used in conjunction to calculate the non-invasive PV loops (*D*). SV, stroke volume; MAP, mean arterial pressure; SW, stroke work.

The image analysis software Segment v.3.1^25^ was used for all image analyses. Group belonging was not blinded during analysis. Left ventricular endocardial contours were manually delineated in all time frames from short-axis images. The resulting time-resolved volume data were used as input for the PV loop algorithm (*Figure 2*). Atrioventricular plane displacement (AVPD) as a biomarker for ventricular long-axis function was measured according to previously published methods.^26^

Infarct size is typically quantified from LGE images; however, *ex vivo* imaging allows for considerably more detailed images and corresponds well with histochemical examinations.^27^ Image stacks from the high-resolution T1-weighted sequence were downsampled to a slice thickness of ∼2 mm. Left ventricular mass was then derived from manually delineated endo- and epicardial contours. The extent of scar and microvascular obstruction (MVO) was manually delineated with aid from *in vivo* LGE, cine images, and photographs of TTC-stained hearts. Oedema was manually traced from CE-SSFP images. Oedema, infarct size, and MVO were expressed as a percentage of total left ventricular mass.

Anaesthetic agent concentration, expiratory CO_2_, heart rate, pulse oximetry, blood pressure, and temperature were logged throughout the experiments. The anaesthetic parameters were continually monitored to achieve stable conditions. Systolic and diastolic blood pressures registered via a femoral artery catheter were inputs to the PV loop algorithm. Data entries nearest in time to the short-axis image acquisition were used for each animal (total time difference 24 ± 46 min).

**Figure 3:**
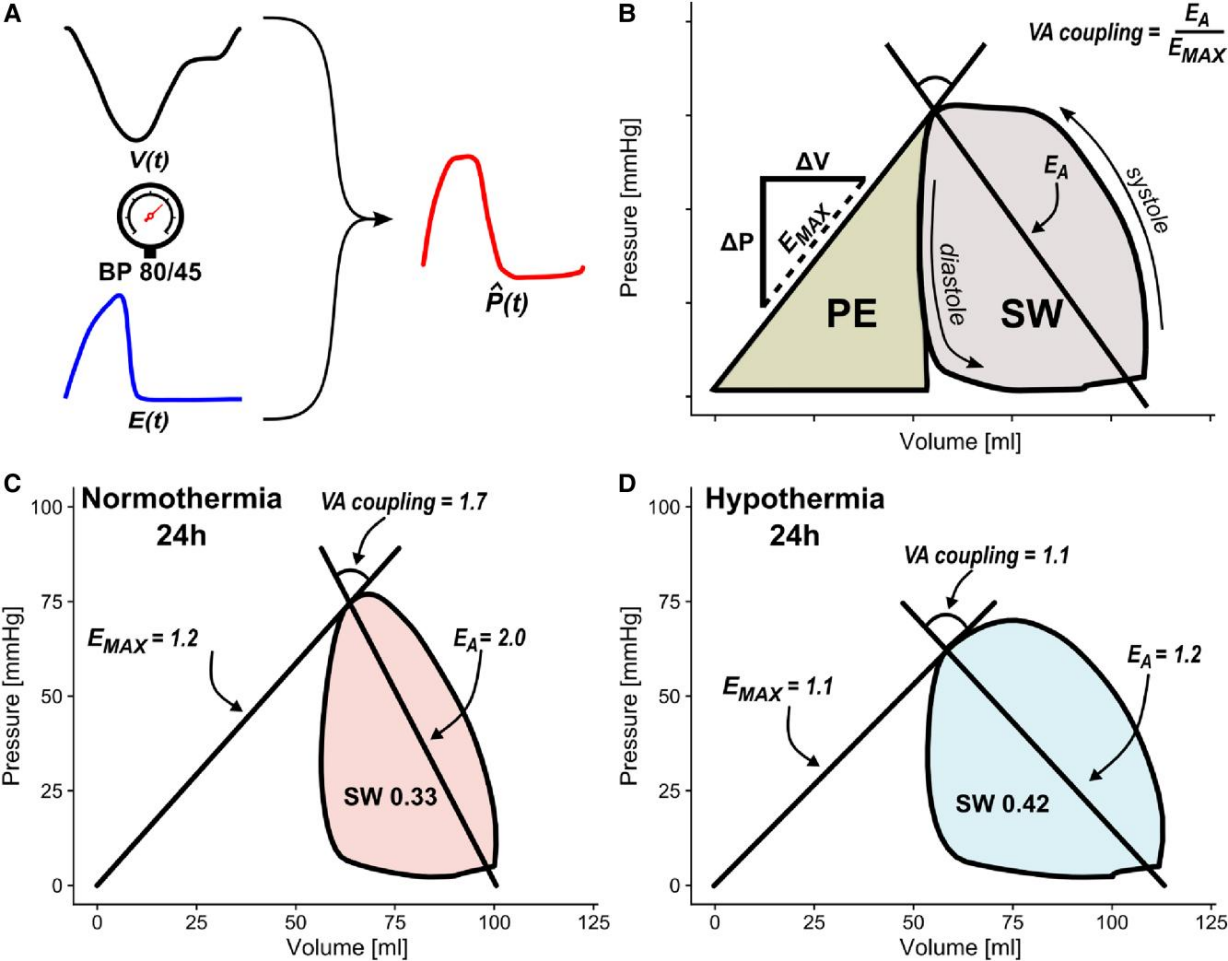
Left ventricular volume curves ‘*V*(*t*),’ systolic and diastolic blood pressures (BP), together with an elastance model ‘*E*(*t*)’ scaled in amplitude and time, yield estimations of left intraventricular pressure curves ‘*P*(*t*)’ (*A*). The subsequent PV loop can be used to estimate information about cardiac energy mechanics (*B*), such as PE, SW, maximal elastance (*E*_MAX_), arterial elastance (*E*_A_), and VA coupling. Elastance is calculated as the pressure change (ΔP) over the volume change (Δ*V*). *Panels C* and *D* portray a typical example from each of the two groups, Normothermia and Hypothermia, at 24 h after ischaemia/reperfusion injury with larger VA decoupling in the Normothermia group compared with Hypothermia.

The PV loop algorithm employed is non-invasive. This means it makes use of the ventricular volume curve derived from CMR together with the peripheral systolic and diastolic blood pressures to render an estimation of the PV loop. The derived leap from discrete peripheral pressures to a continuous ventricular pressure curve is enabled through a general elastance curve scaled in amplitude and time (*Figure 3A*). The original algorithm was previously described in detail by Seeman *et al.*^20^ However, we used an improved model that more physiologically accommodates a broad range of heart rates.^21^ The algorithm models the work exerted by the heart in one heartbeat, known as stroke work (SW), which is defined as the area within the PV loop. The algorithm is available in the event of research collaboration.

A line drawn from the *x–y* intercept, at zero volume and zero pressure, to the point of maximal elastance represents a relatively load-independent measure of contractility labelled *E*_MAX_. The mechanical potential energy (PE) is defined as the area beneath the *E*_MAX_ line. It accounts for the remaining energy stored in the sarcomere at the end of systole that is not dissipated as SW.^28^ The total mechanical energy consumption during a heartbeat equals the combined area of SW and PE, known as PV area (PVA). As the supply and demand of adenosine triphosphate are conditionally in balance in the myocardium, the PVA has been shown to be highly proportional to myocardial oxygen consumption.^29^ This proportionality allows for non-invasive derivations of energy consumption without measuring oxygen *in vivo*. Ventricular efficiency as a percentage can be calculated as SW/(SW + PE)×100. This represents the proportion of the total cardiac energy consumption used to generate stroke volume (SV).

Furthermore, cardiac power output can be calculated as SW × (heart rate/60). The effective arterial elastance (*E*_A_) is defined as the slope from the point of maximal elastance to the point of end-diastolic volume at zero pressure. The ventricle’s interaction with the arterial load is called the ventricular-arterial (VA) coupling, which is defined as the ratio of *E*_A_ over *E*_MAX_ (*Figure 3B*).

### 2.4 Statistics

Differences between groups were compared using the Mann–Whitney *U* test. Differences within the groups were assessed with the Friedman test. Dunn’s test for multiple pairwise comparisons with the Holm correction was used as a *post hoc* test to further assess differences between imaging sessions. *P*-values below 0.05 were considered to indicate significance. Linear regression of the relative change from baseline to 24 h was used to evaluate whether infarct size was the primary driver of cardiac impairment. 24 h was chosen since this was the expected nadir of cardiac impairment. Furthermore, the analysis of covariance (ANCOVA) was used to assess differences between relative changes in PV loop variables adjusted for infarct size. Results in tables and main text are presented as mean ± standard deviation. Plots with error bars show mean ± standard error of the mean, and individual data points are shown as dashed lines. Statistical analyses were performed in R v4.0.3.

## 3. Results

Out of 19 animals, 5 did not complete the study protocol due to ventricular arrhythmia (*n* = 4; two allocated for each group) or failed intervention (*n* = 1) and were not included in the final analysis. Non-invasive PV loops could be measured in all 14 animals that completed the 1-week protocol. The Normothermia group (three females, four males) and the Hypothermia group (two females, five males) did not differ in weight (39.4 ± 3.2 kg vs. 40 ± 2.5 kg; *P* = 0.75).

**Figure 4:**
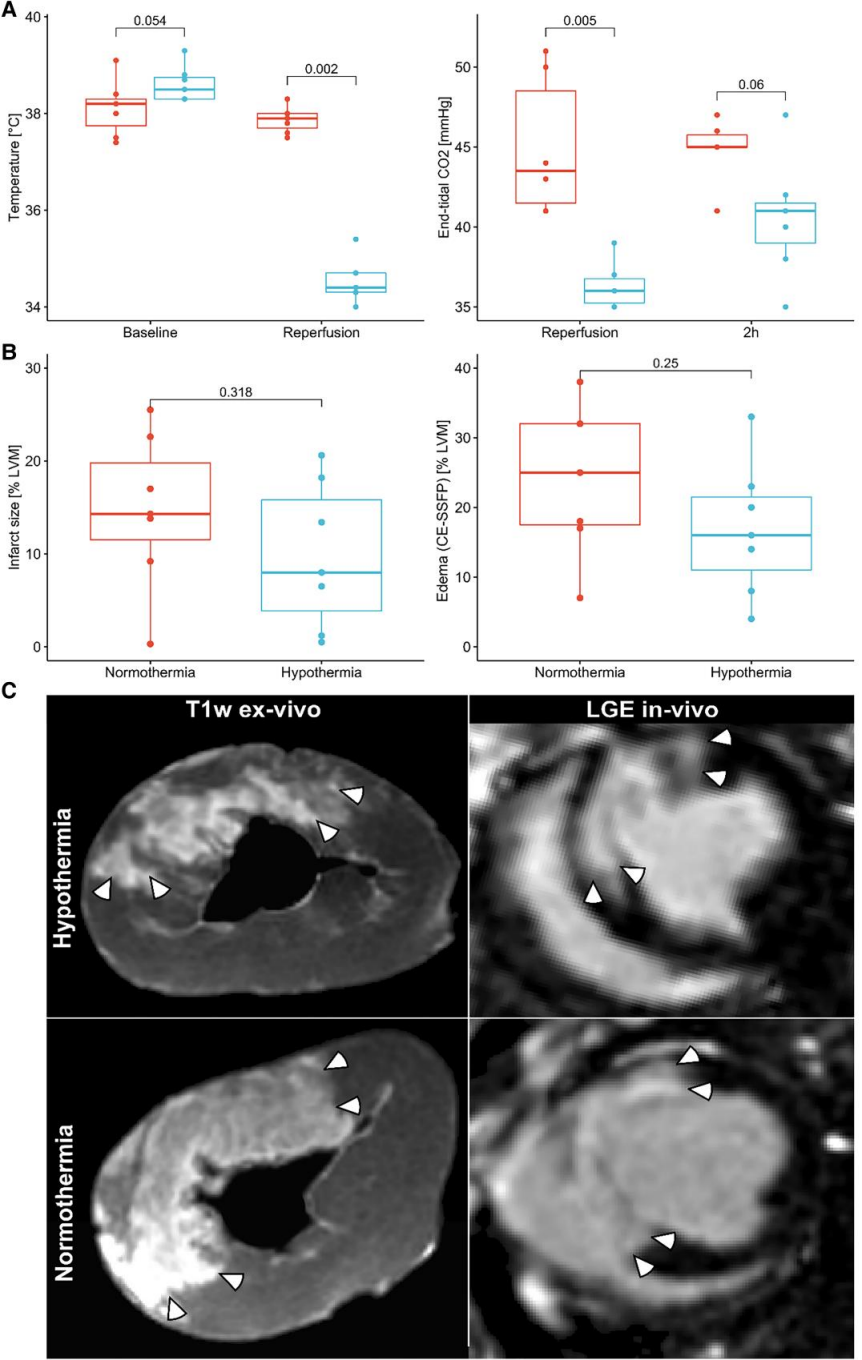
(*A*) The Hypothermia protocol acutely reduced the internal temperature before reperfusion in the Hypothermia group (*n* = 7; rightmost; blue) compared with the Normothermia group (*n* = 7; leftmost; red). Consequently, end-tidal CO_2_ was reduced, which was not fully restored at the 2 h imaging session. (*B*) Box plots showing the infarct size and myocardial oedema for the Normothermia and Hypothermia groups on Day 7. (*C*) Typical examples of T1-weighted *ex vivo* images used for quantification and corresponding *in vivo* LGE images used for the Hypothermia and Normothermia groups. White arrows demarcate hyperintense regions indicating infarct. CE-SSFP: contrast-enhanced steady-state free precession; LVM: left ventricular mass.

The temperature at reperfusion was 37.9 ± 0.3°C in the Normothermia group and 34.5 ± 0.5°C in the Hypothermia group (*P* = 0.002; *Figure 4*). End-tidal carbon dioxide (etCO_2_) at reperfusion was 36.3 ± 1.5 mmHg in the Hypothermia group and 45.0 ± 4.4 mmHg in the Normothermia group (*P* = 0.005; *Figure 4*). The lower etCO_2_ seen in the Hypothermia group could also be detected to some extent 2 h post-reperfusion (*P* = 0.06; *Figure 4*).

### 3.1 Infarct size and myocardium at risk

Ischaemia and reperfusion caused infarct sizes of 10 ± 8% in the Hypothermia group compared with 15 ± 8% in the Normothermia group (*P* = 0.32; *Figure 4*). The myocardial oedema was 17 ± 10% and 24 ± 11% in the Hypothermia and Normothermia groups (*P* = 0.25), respectively. This amounted to an estimated myocardial salvage of 52 ± 26% and 41 ± 33%, respectively, for the Hypothermia and Normothermia groups (*P* = 0.32). The MVO was similar for both groups (Hypothermia: 2.0 ± 3.1%; Normothermia: 2.2 ± 2.3%; *P* = 0.70).

### 3.2 Ventricular volumes and pressures

**Figure 5:**
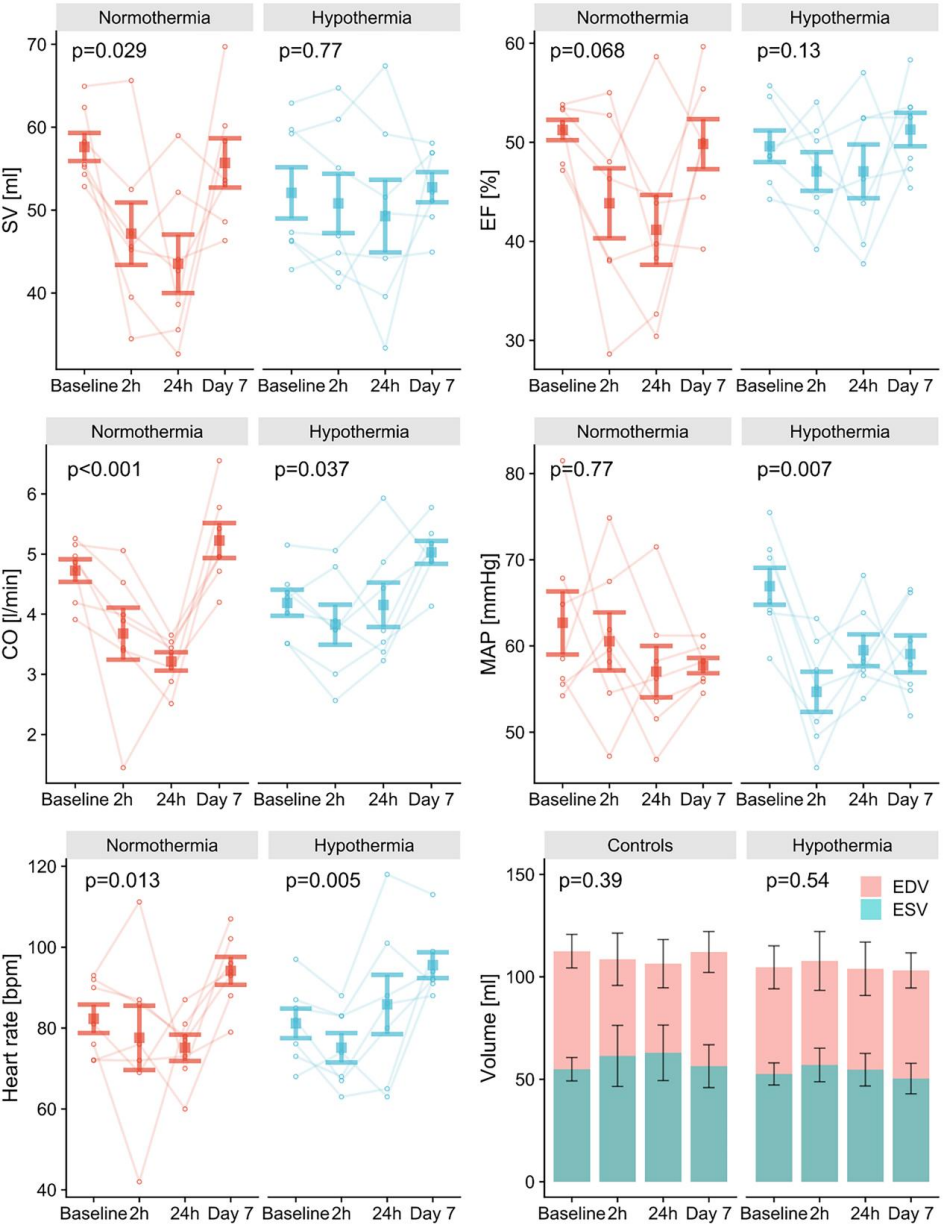
Error bar plots showing mean ± standard error of the mean for SV, EF, CO, MAP, heart rate, end-diastolic volume (EDV), and end-systolic volume (ESV). The reductions in volumetric variables seen in the Normothermia group (*n* = 7) were generally ameliorated in the Hypothermia group (*n* = 7). MAP fell significantly in the Hypothermia group after the intervention of mild hypothermia. *P*-values from Friedman’s test compare results over four time points within each group.

Volumetric and PV loop measurements are presented in *Table 1*. The Hypothermia group displayed less pronounced reductions in SV (−6%; Friedman’s test: *P* = 0.77), ejection fraction (EF: −6%; *P* = 0.13), cardiac output (CO: −0%; *P* = 0.037), and AVPD (−9%; *P* = 0.51) at 24 h compared with the Normothermia group (SV: −24%, *P* = 0.029; EF: −20%, *P* = 0.068; CO: −32%, *P* < 0.001; AVPD: −28%, *P* = 0.01; *Figure 5* and Supplementary material online, *Figure S2*). The animals subjected to hypothermia showed an acute reduction in mean arterial pressure (MAP) at 2 h from baseline (−18%; Dunn’s test: *P* = 0.007), which recovered and was not significantly decreased at 24 h (Dunn’s test: *P* = 0.19), whereas MAP was stable in the Normothermia group throughout all measurements (*P* = 0.77). Heart rate did not differ between groups (*P* = 0.72) but showed an increase on Day 7 compared with 2 h (Hypothermia +28%; Dunn’s test: *P* = 0.013) and 24 h (Normothermia +25%; Dunn’s test: *P* = 0.027) post-reperfusion.

**Table 1:** Table of volumetric, infarct, and pressure-volume loop characteristics.

	Baseline		2 h		24 h		Day 7	
Variables	Normothermia	Hypothermia	Normothermia	Hypothermia	Normothermia	Hypothermia	Normothermia	Hypothermia
EDV (mL)	110 ± 8.2	100 ± 10	110 ± 13	110 ± 14	110 ± 12	100 ± 13	110 ± 10	100 ± 8.5
ESV (mL)	55 ± 5.7	53 ± 5.4	61 ± 15	57 ± 8.2	63 ± 14	55 ± 7.9	56 ± 10	50 ± 7.4
SV (mL)	58 ± 5	52 ± 8	47 ± 10	51 ± 10	44 ± 9	49 ± 12	56 ± 8	53 ± 5
EF (%)	51 ± 3	50 ± 4	44 ± 9	47 ± 5	41 ± 9	47 ± 7	50 ± 7	51 ± 5
CO (l/min)	4.7 ± 0.5	4.2 ± 0.6	3.7 ± 1.1	3.8 ± 0.9	3.2 ± 0.4	4.2 ± 1.0	5.2 ± 0.8	5.0 ± 0.5
AVPD (mm)	11.5 ± 1.5	11.3 ± 1.5	8.5 ± 1.0	10.4 ± 2.0	8.7 ± 2.4	10.1 ± 2.8	10.2 ± 1.4	10.6 ± 1.4
Heart rate (bpm)	82 ± 9	81 ± 10	78 ± 21	75 ± 10	75 ± 9	86 ± 19	94 ± 9	96 ± 8
Infarct size T1-w (%LVM)							15 ± 8	10 ± 8
Oedema CE-SSFP (%LVM)							24 ± 11	17 ± 10
MVO (%LVM)							2 ± 2	2 ± 3
Myocardial salvage (%)							41 ± 33	52 ± 26
MAP (mmHg)	63 ± 10	67 ± 6	61 ± 9	55 ± 6	57 ± 8	59 ± 5	58 ± 2	59 ± 6
SW (J)	0.50 ± 0.11	0.49 ± 0.10	0.39 ± 0.12	0.39 ± 0.13	0.32 ± 0.13	0.38 ± 0.10	0.46 ± 0.08	0.42 ± 0.05
PE (J)	0.28 ± 0.05	0.30 ± 0.05	0.31 ± 0.08	0.27 ± 0.06	0.30 ± 0.05	0.27 ± 0.07	0.28 ± 0.05	0.24 ± 0.05
Efficiency (%)	64 ± 3	62 ± 5	55 ± 11	58 ± 7	51 ± 11	59 ± 8	62 ± 7	64 ± 5
External Power (W)	0.71 ± 0.16	0.69 ± 0.15	0.53 ± 0.20	0.51 ± 0.18	0.41 ± 0.11	0.56 ± 0.16	0.74 ± 0.12	0.70 ± 0.10
*E*_MAX_ (mmHg/mL)	1.3 ± 0.2	1.5 ± 0.2	1.2 ± 0.4	1.2 ± 0.2	1.1 ± 0.4	1.2 ± 0.1	1.3 ± 0.2	1.3 ± 0.2
*E*_A_ (mmHg/mL)	1.4 ± 0.1	1.8 ± 0.3	1.9 ± 0.4	1.5 ± 0.3	1.9 ± 0.3	1.7 ± 0.5	1.5 ± 0.3	1.5 ± 0.2
Ventricular-arterial Coupling	1.1 ± 0.1	1.2 ± 0.2	1.6 ± 0.7	1.3 ± 0.3	1.8 ± 0.6	1.4 ± 0.4	1.3 ± 0.4	1.1 ± 0.2

CO: cardiac output; EDV: end-diastolic volume; ESV: end-systolic volume; SV: stroke volume; EF: ejection fraction; bpm: beats per minute; T1-w: T1-weighted; LVM: left ventricular mass; CE-SSFP: contrast-enhanced steady-state free precession; MAP: mean arterial pressure; MVO: microvascular obstruction; *E*_MAX_: ventricular elastance; *E*_A_: Arterial elastance; PE: potential energy; SW: stroke work.

### 3.3 Cardiac energy and mechanics

**Figure 6:**
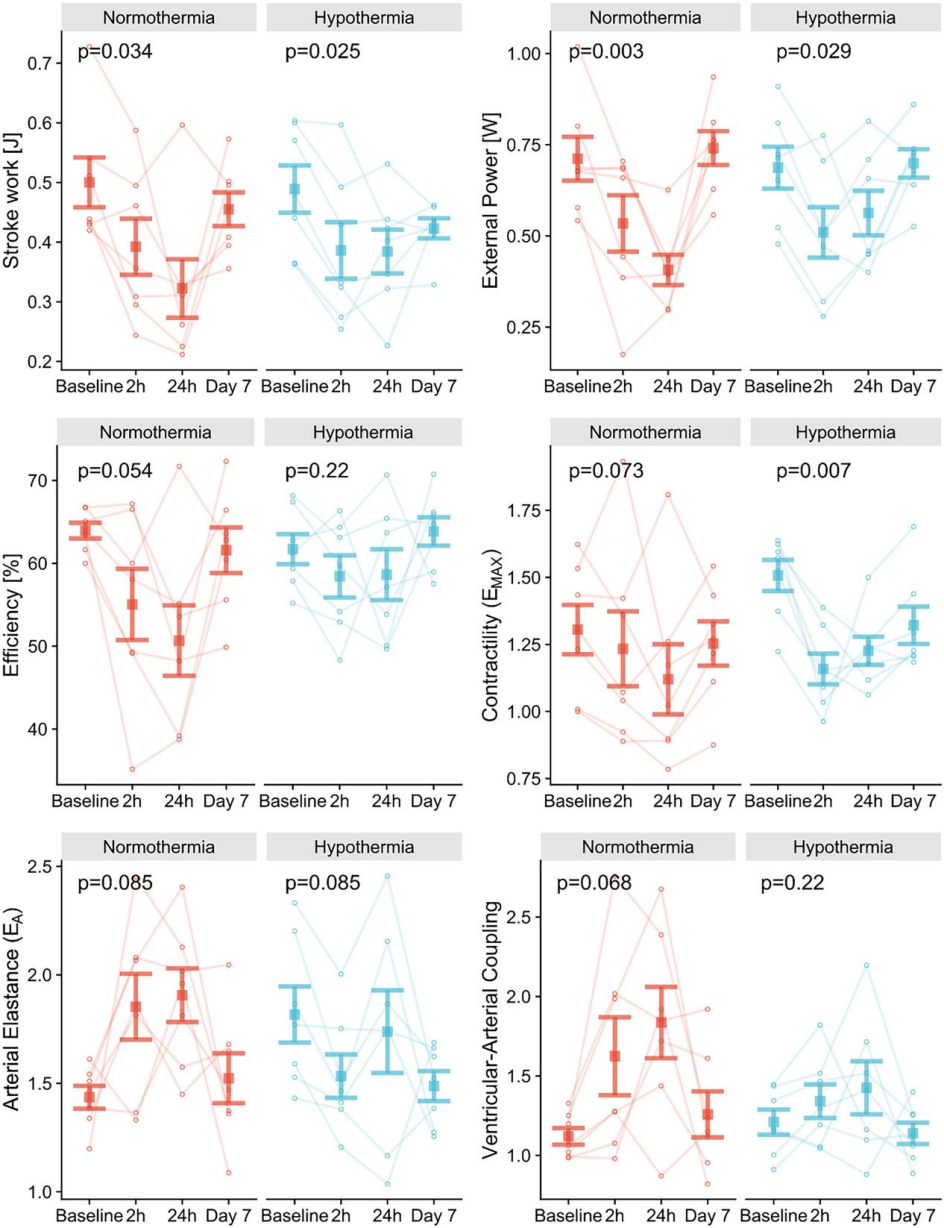
Error bar plots showing mean ± standard error for SW, external power, efficiency, contractility (*E*_MAX_), arterial elastance (*E*_A_), and VA coupling. The adverse effects of ischaemia/reperfusion injuries on PV loop-derived variables seen in the Normothermia group (*n* = 7) were generally attenuated in the Hypothermia group (*n* = 7). *P*-values from Friedman’s test compare results over four time points within each group.

The adverse effects of ischaemia/reperfusion injuries on PV loop-derived variables seen in the Normothermia group were generally attenuated in the Hypothermia group (*Figure 6*). Peak reductions in SW and external power were smaller in the Hypothermia group (−22%, *P* = 0.025; −19%, *P* = 0.029) than in the Normothermia group (−36%, *P* = 0.034; −42%, *P* = 0.007). Additionally, ventricular efficiency was preserved in the Hypothermia group (*P* = 0.22), whereas it declined in the Normothermia group with a nadir at 24 h and was followed by partial recovery (*P* = 0.054). However, no trends or differences within or between groups were seen for PE.

The Hypothermia group displayed a larger baseline *E*_MAX_ than the Normothermia group (1.5 ± 0.2 vs. 1.3 ± 0.2 mmHg/mL, *P* = 0.051). However, *E*_MAX_ sharply declined at 2 h in the Hypothermia group (−23%; Dunn’s test: *P* = 0.009), likely attributable to the sharp drop in MAP at the same time. This was not seen in the Normothermia group (−6%; *P* = 0.073). The Hypothermia group also showed higher baseline arterial elastance than the Normothermia group (1.8 ± 0.3 vs. 1.4 ± 0.4 mmHg/mL; *P* = 0.02), driven primarily by MAP. In contrast, the difference was reversed at 2 h, with lower arterial elastance in the Hypothermia group, although not significant (*P* = 0.21). Furthermore, VA coupling was more consistent in the Hypothermia group with a peak at 24 h (+17%; *P* = 0.22), whereas the Normothermia group’s VA coupling was inflated (+64%; *P* = 0.068), possibly due to a higher afterload in normothermic animals. Of note, one animal with minimal infarct size (<1%) differed from the rest of the Normothermia group in several variables, especially at 24 h. There were no statistical differences for volumetric or PV loop-derived variables remaining on Day 7.

### 3.4 Mild hypothermia modulates the effect of infarct size on functional impairment

**Figure 7:**
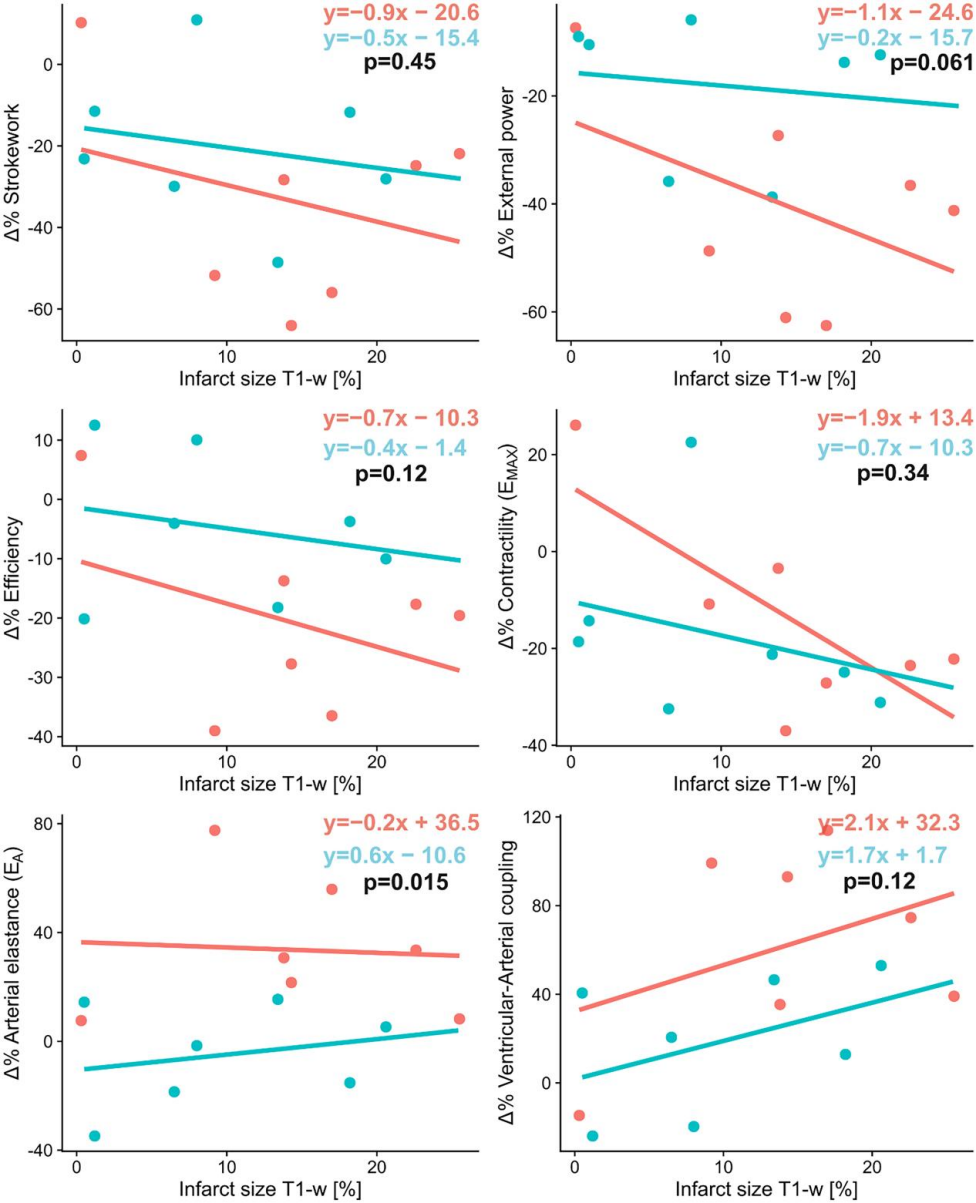
Regression analyses of the relative changes from baseline to 24 h after ischaemia/reperfusion with six PV loop-derived measurements. If the regression lines differ in slope or offset between the Hypothermia group (*n* = 7; bottom equation; blue) and Normothermia group (*n* = 7; top equation; red) that means that hypothermia itself, in addition to the infarct size, modulates the observed results. There are slight trends in external power, efficiency, *E*_MAX_, and VA coupling. This means that the intervention of mild hypothermia offsets and attenuates the adverse effects caused by ischaemia/reperfusion. *P*-values by the ANCOVA tests if the relative reductions in PV loop variables between Hypothermia and Normothermia groups differ while adjusting for infarct size.

The relative changes of cardiac energetic variables from baseline to 24 h were plotted against infarct size to investigate whether the effects of infarct size differ irrespective of hypothermia or not. The increase in arterial elastance and, to a borderline extent, the decrease in external power were significantly modulated by hypothermia (ANCOVA: *P* = 0.015; *P* = 0.061; *Figure 7*). However, the collective trend of the PV loop variables demonstrates energetically more advantageous conditions in the Hypothermia group, such as higher efficiency and lower VA coupling, compared with the Normothermia group at 24 h when adjusting for infarct sizes.

## 4. Discussion

For the first time, this study presents the effects of mild hypothermia (<35°C) on ischaemia/reperfusion injuries with non-invasive PV loops. Mild hypothermia upheld cardiac performance in the acute and subacute phases as indicated by trends towards more preserved SV, EF, AVPD, ventricular efficiency, and more favourable VA coupling, compared with normothermia. Furthermore, the results indicate that hypothermia has cardioprotective properties not fully explained through infarct size differences. The mechanism could, at least partly, be the result of lower mean arterial blood pressure from hypothermia in the acute phase.

### 4.1 Relation to earlier studies

There are two novel designs of this study. First, we serially investigated the temporal pattern of myocardial PV loop variables after ischaemia/reperfusion in an experimental setup throughout the first week. Consequently, baseline values are available, which, for natural reasons, are not available in human studies. Secondly, we used a novel non-invasive PV loop method to explore alterations in cardiac performance.

We followed a similar ischaemia/reperfusion protocol as Götberg *et al.*^2,23,30^ but extended the protocol to four CMR imaging sessions to assess the ventricular function throughout the first week. While earlier experimental hypothermia studies focused on infarct size^3^ or the timing of cooling,^2^ we instead focused on the temporal changes in global ventricular energetics and mechanics with PV loops over 1 week. The significant decrease in infarct size and myocardial salvage by mild hypothermia intervention in the study by Götberg *et al*.^23^ could not be replicated. Possible explanations for this are, for example, outliers and differences in the anaesthetic protocol. A likely reason for the smaller infarcts in our study is that we recorded significantly lower blood pressures (MAP: ∼60 vs. ∼100 mmHg) and heart rates (∼80 vs. ∼100 bpm) at reperfusion compared with the animals from the prior experiments.^30^

The main findings in this study are that the animals subjected to hypothermia at reperfusion trended towards more preserved cardiac energetics than normothermic animals. The regression analyses indicate that the reductions in PV loop variables were in part attributable to smaller infarct sizes as shown earlier,^23^ but also that hypothermia modulated the adverse effects on contractile properties and energetics of ischaemia/reperfusion in non-infarcted myocardium during the first 24 h. The more preserved ventricular volumes as well as long-axis function in the Hypothermia group suggests that mild hypothermia attenuated myocardial stunning. This would support prior experimental work that showed superior contractile recovery of injured myocardial segments with mild hypothermia than ischaemic preconditioning at normothermia.^31^

In this study, a possible mechanism of cardioprotection could be a drop in blood pressure in the acute phase since lowering blood pressure is cardioprotective because of reduced pressure work and wall tension needed to sustain adequate circulation. Lower blood pressure, in addition to more preserved ventricular volumes, would unload the LV from excessive work. While a drop in arterial pressure is beneficial from a cardio-centric perspective of reduced afterload, it could elicit transient renal failure. Blood pressure after an induced hypothermic state has been reported to be unchanged or increased,^2^ although accounts of transiently increased or reduced blood pressure have also been described.^32^ One explanation for this variability can be differing anaesthetic depths, as especially topical cooling, which some studies have utilized, can exert a strong stimulus in a conscious individual. Shivering, which is a part of the sympathetic response to combat hypothermia, indicates an intense sympathetic drive and often coincides with increased MAP. Blood pressure, however, decreased as a result of cooling in our study. In this specific case of rapid hypothermia induction, the cold fluid will likely increase preload and cooling will reduce arterial blood pressure and hence afterload. The reduction in blood pressure caused by calcium channel blockers or nitrates and cooling likely have similar effects on afterload. To this, the effect of decreased metabolic demand from cooling should be added. While it is beneficial to keep blood pressure tolerably low in clinical interventions of STEMI, potential synergetic effects from clinically preventing increased blood pressure during induced hypothermia remain speculative.

### 4.2 Physiologic relevance

The Hypothermia group indicated a more efficient transfer of work and energy between the ventricle and the arterial tree. The haemodynamic synthesis of VA coupling was initially developed by Sunagawa *et al.*^33^ This concept characterizes the relation between the arterial tree and LV as two elastic ‘chambers’ and uses the same units. Furthermore, it was predicted and experimentally demonstrated^34^ that optimal VA coupling, that is, energy transfer between the ventricle and arterial tree without excessive changes in pressure, is ideal when the VA coupling is near 1. Ratios of *E*_A_/*E*_MAX_ between 0.6 and 1.2 have been found in humans,^35^ in feline,^36^ and in canine hearts^37^ at rest. Our baseline values for *E*_A_/*E*_MAX_ (1.16 ± 0.18) agree with earlier reports.

During intense exercise, VA coupling decreases due to a relative increase in contractility ‘*E*_MAX_,’ and the ratio *E*_A_/*E*_MAX_ has been reported to fall over 50%.^38,39^ In contrast, severe afterload mismatch is present in systolic heart failure, which can be seen in VA coupling ranging between 1.3 and 4.3.^40^ Our results indicate that the Normothermic group (1.80 ± 0.59) more closely resembles systolic heart failure at 24 h than the Hypothermia group (1.4 ± 0.44) and shows less favourable energetics, including measures of efficiency, work, and power.

### 4.3 Hypothermia and cardioprotection

Clinical and preclinical trials have shown conflicting results regarding hypothermia as an effective cardioprotective therapy.^3,7^ Hypotheses of the lack of successful trials have concerned the efficacy of rapid cooling or extension of the time-to-treatment. Our results indicating improved energetics and mechanics after ischaemia/reperfusion in the Hypothermia group add insights into the cardioprotective effects of hypothermia. Such effects are likely also present in clinical studies but have not yet been studied to the best of our knowledge.

In the same manner, as fever increases the body’s metabolic demands, a reduction of the internal temperature decelerates the cellular enzymatic machinery and may, therefore, slow the ischaemic myocyte damage.^41^ Several mechanisms of cardioprotection by hypothermia have been proposed, for example, increased resistance to mitochondrial oxidative stress,^42^ increased cellular structural integrity,^43^ and decreased apoptosis.^44^ As it currently stands, cardiovascular drugs have a low likelihood of gaining approval for clinical use (∼7%) after transitioning from experimental studies.^45^ The apparent advantage of hypothermia might be the ‘catch-all’ mechanism of enzymatic deceleration that involves known and unknown pathways for reducing ischaemic injuries, and as the current results indicate, unload the heart from unnecessary strain.

In this study, the infarcts in the Hypothermia group were 10 ± 8% compared with 15 ± 8% for the Normothermia group, which is in line with earlier studies although the decrease in size was not statistically significant. Even if the hypothermic animals were subjected to 5 min longer periods of ischaemia, as well as exhibiting unexplained and offsetting trends towards higher baseline temperatures and MAP, they presented with more favourable energetic and mechanical states after reperfusion. This suggests that hypothermia protects from the adverse effects of reperfusion, at least in the acute and subacute phases. These findings extend the knowledge about the cardioprotective mechanisms of hypothermia beyond limiting infarct size.

### 4.4 Clinical relevance

PV loops are useful for visually displaying various aspects of cardiac performance, such as contractility, afterload, preload, energetics, or lusitropy, and have been used to analyse the effects of various cardiac interventions.^28^ As stated by previous studies,^46,47^ the theoretical advantage of VA coupling over left ventricle ejection fraction (LVEF) lies in examining the individual components of *E*_A_/*E*_MAX_ to discern if the haemodynamic alterations are due to properties of the LV, arteries, or both. It has been demonstrated that the VA coupling is strongly predictive of adverse outcomes,^48,49^ and it has been reported to have a higher predictive value than LVEF in haemodialysis patients.^50^ While invasive PV loops constitute the ‘gold standard,’ non-invasive methods, such as the one currently employed, provide similar information and can be used in more patient groups and in more extensive studies to study pathophysiology, guide treatment, evaluate new therapies, or predict prognosis in a broader set of cardiac diseases.

### 4.5 Limitations

Generalizing these results to humans should be made with some care and our sample sizes primarily lend our results to hypothesis-generating purposes. Examples of limiting factors are that most patients are not sedated when treated for ischaemia—although sedations are frequently used during therapeutic hypothermia; in addition, patients are older and often have comorbidities such as long-standing hypertension, diabetes, atherosclerosis, or prior MI; and patients are treated with other medications not included in our study.

The time difference between short-axis acquisition from magnetic resonance imaging (MRI) and blood pressure measurement (24 ± 46 min) could result in smoothing of the results, i.e. diminishing the findings. However, the anaesthesia protocol, including drug administration and ventilator settings, was tightly controlled to minimize alterations.

The LV end-diastolic pressure (LVEDP) is not measured but chosen by the user, and hence the bottom right corner of the PV loop is an estimation which is a limitation of the method. We kept the LVEDP at 5 mmHg for all time points. By doing so, we did not add systematic biases. After the ischaemia/reperfusion injuries, the most likely outcome would be acute increases in LVEDP, accentuating differences between time points. A sensitivity analysis of varying LVEDP between 0 and 15 mmHg was carried out in the original study.^20^ For example, ventricular efficiency and SW maximally deviated 3 and 10% from the model mean, respectively, and user-determined LVEDP was, therefore, considered to have a low impact on the overall results.

Finally, we have not included *in vivo* comparisons in our study as it has been published earlier. Seemann *et al*.^20^ describes the original algorithm, mathematical equations in detail, and *in vivo* validation. We use an updated algorithm that also has been compared with *in vivo* measurements.^21^ The agreement was excellent with invasive data, evident in differences for SW (0.00 ± 0.03 J), ventricular efficiency (0.1 ± 0.4%), and ventricular elastance (0.1 ± 0.1 mmHg/mL).

## 5. Conclusion

Inducing mild hypothermia before reperfusion indicated improved cardiac efficiency and more favourable VA coupling during the first week after ischaemia/reperfusion injury as measured by non-invasive PV loops by CMR. The results suggest that hypothermia has cardioprotective properties not fully explained by infarct size differences. The pathophysiological mechanism of cardioprotection may partly be due to an acute reduction in arterial afterload, unloading the injured LV during the initial days after ischaemia/reperfusion.

## Supplementary Material

cvad028_Supplementary_Data

## Notes

Translational perspective Knowledge of the cardioprotective effects of hypothermia remains incomplete due to prior studies lacking serial observations and overlooking global cardiac dysfunction. Therefore, we analysed the effects of hypothermia in ischaemia/reperfusion on cardiac energy mechanics by non-invasive pressure–volume (PV) loops for 1 week. The cooling intervention (mean 34.5°C) significantly ameliorated cardiac impairment compared with normothermia. Hypothermia yielded favourable energy mechanics, such as higher efficiency and better VA coupling, compared with normothermia, and suggested to protect cardiac function incremental to reducing infarct size. In addition, non-invasive PV loops by CMR could be used clinically and help elucidate cardiovascular haemodynamic mechanisms in future studies.
