# Supplementary material for: Mild hypothermia attenuates ischaemia/reperfusion injury: insights from serial non-invasive pressure–volume loops
Source: Cardiovasc Res. 2023 Feb 3;119(12):2230–43. doi: 10.1093/cvr/cvad028 (PMC10578916; doi:10.1093/cvr/cvad028)
Supplement: cvad028_Supplementary_Data [file cvad028_supplementary_data.docx]

**Appendix**

**Supplementary figures**

**
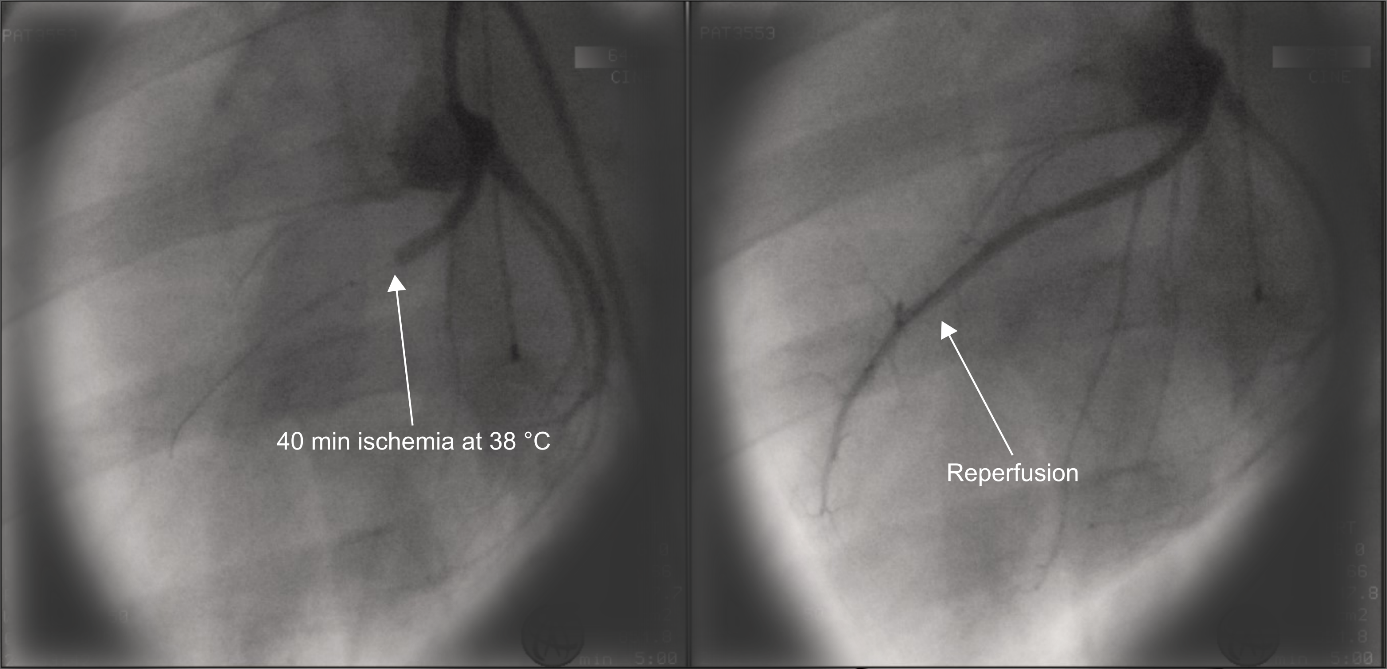
**

**Figure S1.**  An example of the ischemia/reperfusion procedure on fluoroscopy imaging. The balloon was inflated in the left descending artery for all animals.

**
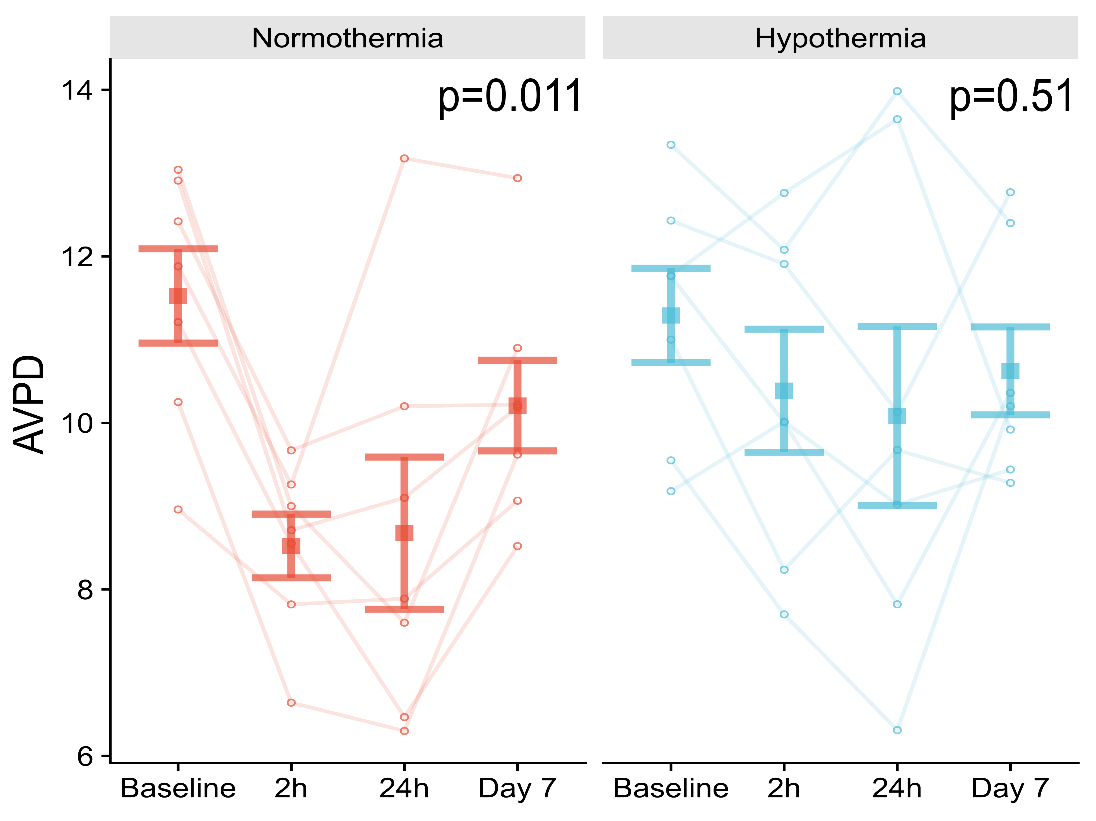
**

**Figure S2.** Error bar plot of showing mean ± standard error of the mean for atrioventricular plane displacement (AVPD). AVPD as a marker of ventricular function was more decreased in the Normothermia group in the acute phase than the Hypothermia group. P values from Friedman’s test comparing results over four time points within each group.
